# Testing methods used to predict disease progression in children with early‐stage type 1 diabetes: A systematic review and meta‐analysis

**DOI:** 10.1111/dme.70077

**Published:** 2025-05-28

**Authors:** Rabbi Swaby, Kruthika Narayan, Claire Scudder, Julia Townson, Richard A. Oram, Kirstine J. Bell, Maria E. Craig, Colin Dayan, Paul Aveyard, Rachel E. J. Besser

**Affiliations:** ^1^ Centre for Human Genetics, Nuffield Department of Medicine, NIHR Oxford Biomedical Research Centre University of Oxford Oxford UK; ^2^ Charles Perkins Centre and Faculty of Medicine and Health University of Sydney Sydney New South Wales Australia; ^3^ Institute of Endocrinology and Diabetes The Children's Hospital at Westmead Westmead New South Wales Australia; ^4^ Centre for Trials Research Cardiff University Cardiff UK; ^5^ NIHR Exeter Biomedical Research Centre University of Exeter Exeter UK; ^6^ Paediatrics and Child Health UNSW Medicine Sydney New South Wales Australia; ^7^ Clinical Diabetes and Metabolism Cardiff University, School of Medicine Cardiff UK; ^8^ Nuffield Department of Primary Care Health Sciences University of Oxford Oxford UK; ^9^ Department of Paediatrics John Radcliffe Hospital Oxford UK; ^10^ NIHR Oxford Health Biomedical Research Centre Warneford Hospital Oxford UK

**Keywords:** autoimmunity, children and adolescents, prediction of diabetes, type 1 diabetes

## Abstract

**Aims:**

Current guidance on how best to monitor children and young people (CYP) with early‐stage type 1 diabetes is evidenced mainly by expert consensus. This systematic review and meta‐analysis aims to evaluate the current evidence for tests used to predict disease progression.

**Methods:**

Data were sourced from PubMed, Cochrane Central, Ovid Embase and Scopus. The association (hazard ratio [HR]) between test positivity and progression to stage 3 type 1 diabetes in CYP aged ≤18 years with ≥2 islet autoantibodies was examined. Data were pooled using random effects models, and the Hartung–Knapp–Sidik–Jonkman (HKSJ) method was used to adjust confidence intervals to account for greater uncertainty. The risk of bias was evaluated using the QUADAS‐2 tool (CRD42023393960).

**Results:**

In this study, 12,923 studies were identified and 285 underwent full‐text review. Thirty‐four studies (*n* = 6866 CYP, median age 11.8 years [IQR, 6.6–13.8]) were included. Overall, 2080 (30%) CYP progressed to stage 3 type 1 diabetes over a median follow‐up of 5 years (IQR 2–5). The pooled HR for tests that predicted progression were: 1.40 (95% CI 1.07–1.84) for fasting glucose (OGTT), 3.19 (1.75–5.82) for 2‐h glucose (OGTT), 6.43 (1.21–34.18) for the M120 above the median value, 3.12 (2.19–4.43) per 1‐unit increase in Index 60 and 1.40 (1.17–1.68) per 1.1 mmol/mol increase in HbA1c (C‐statistics 0.7–0.8). Evidence for other tests, including CGM, was uncertain.

**Conclusions:**

The OGTT, its related tests (M120, Index60) and HbA1c predict progression to stage 3 in CYP with early‐stage type 1 diabetes. Other tests, including CGM, need more evidence to support their use as predictive tests in this context.

**What's new?**

- The development of disease modifying therapies to prevent or delay disease progression has emphasised the need for more robust evidence on the tests used to monitor individuals with early‐stage type 1 diabetes.
- The oral glucose tolerance test (OGTT), M120, Index60 and HbA1c predict progression, but more evidence is needed for other tests, including continuous glucose monitoring, IVGTT and C‐peptide.
- The OGTT, M120, Index60 and HbA1c are useful tests to monitor children for progression to stage 3 type 1 diabetes.

## 1 INTRODUCTION

General population screening programs that identify children and young people (CYP) with early‐stage type 1 diabetes are increasing worldwide. These identify individuals with two or more islet autoantibodies (IA) with normoglycaemia (stage 1), dysglycaemia (stage 2), or a blood glucose above the diagnostic threshold of ≥11.1 mmol/L (stage 3).^1^ An accompanying follow‐up pathway is needed to see the benefits of screening, including a reduction in the rate of hospitalisation and diabetic ketoacidosis (DKA) at diagnosis and to identify individuals eligible for preventative therapies.^2^, ^3^, ^4^, ^5^

The oral glucose tolerance test (OGTT) is the current gold standard method to stage and monitor disease progression.^6^ Other tests are available to assess beta‐cell function, directly or indirectly, but uncertainty remains about their predictive value. A recent international consensus recognised the need to offer less invasive testing, suggesting practical alternatives to support monitoring in clinical care.^7^ This study aims to synthesize the evidence on the value of tests to predict progression to stage 3 type 1 diabetes in children with ≥2 IA.

## 2 METHODS

### 2.1 Search strategy and selection criteria

Preferred Reporting Items of Systematic Reviews and Meta‐analyses (PRISMA) guidelines were used and a PROSPERO registered protocol, CRD 42023393960.^8^ PubMed, Cochrane Central, Ovid Embase and Scopus were searched, from January 1974, when IA were first described,^9^ to 07 August 2024, with no language restrictions. Clinical trial registries were searched, and authors contacted for data clarification. The search strategy combined relevant thesaurus terms for PubMed and Ovid Embase and sensitive free‐text terms and phrases, truncated as necessary, using an adapted search filter to identify paediatric studies (Table S1).^10^

Studies were included if they reported on CYP with ≥2 IA being assessed with a test to detect disease progression. Studies were excluded that reported on populations whose median age was >18 years, single IA status or who were known to have stage 3 type 1 diabetes at baseline. The value of the test in predicting progression to stage 3 type 1 diabetes if testing positive compared with negative was assessed, and test methods were tabulated to provide a brief overview (Table S2).

Studies were assessed for eligibility independently by authors (RS, CS or KN), against inclusion/exclusion criteria, on title, abstract and full texts, using Covidence software.^11^ Disagreement on study selection was resolved between the review authors (RS, CS or KN) or the whole review team.

### 2.2 Data analysis

Data were extracted by one author (RS) and checked by a second (KN). This included first author, publication year, study design, age at study entry, follow‐up duration, region, population type, autoantibodies tested, predictive tests and thresholds, primary (hazard ratio [HR]) and secondary summary outcome measures with 95% confidence intervals (sensitivity, specificity, positive predictive value [PPV], negative predictive value [NPV], area under the curve [AUC]). Standard errors were calculated from confidence intervals or their logHR and *p*‐values if necessary (Table S3).^12^ Studies that presented Kaplan–Meier curves had HRs calculated from graphical data.^13^, ^14^

Studies were assessed for risk of bias independently by authors (RS and KN) using the QUADAS‐2 tool.^15^ Any studies with concerns in the index test domain were rated high risk.

Studies that applied similar tests were pooled. When different thresholds were used across similar tests, HRs and variances were inverted or rescaled to define test positivity by a common threshold, assuming the test variable followed a normal distribution using standard formulae (Table S3).^16^

Several studies included the same population. To ensure each meta‐analysis included the same participant once, a hierarchy of criteria determined which study was selected for each analysis: (1) low risk of bias; (2) most recent publication; (3) larger sample size. Studies assessing one test using different thresholds in the same population were included in leave‐one‐out analyses. When studies combined multiple datasets, each was treated separately.

A random‐effects meta‐analyses were conducted using the metagen package (R, version 4.2.2), to give a pooled estimate for each test. For analyses containing fewer than 10 studies, the Hartung–Knapp–Sidik–Jonkman (HKSJ) method was used to adjust confidence intervals to account for greater uncertainty. If this adjustment resulted in confidence intervals narrower than the classic random effects model, an ad‐hoc correction was applied.^17^ Levels of heterogeneity were judged to be low (*I* ^2^ < 25%), moderate (*I* ^2^ 25%–50%) or high (*I* ^2^ > 50%).^12^ Meta‐analyses with 3 or more studies and an *I* ^2^ of more than 30% had 95% prediction intervals calculated, to demonstrate the likely outcome in a similar future study.^18^ Where meta‐analysis proved inappropriate, the results were summarised narratively.

Subgroup analyses were conducted to assess the effect of study characteristics that could impact the pooled estimate and level of heterogeneity in each meta‐analysis. These included the use of islet‐cell antibody (ICA; which confirms the presence of any IA but does not identify individual antibodies) as a marker of early‐stage type 1 diabetes, the use of an intervention, risk of bias, risk period and population type. The significance for subgroup analyses was set at *p*‐value ≤0.01.

## 3 RESULTS

### 3.1 Study selection and characteristics

We identified 12,923 studies (9328 from the initial search, and 3595 studies from the citation search) with 5264 studies screened by title and abstract after removing duplicates (*n* = 7659). Two hundred and eighty‐five studies underwent full‐text review and 34 were included in the final analyses, once ineligible papers were removed (*n* = 4979) (Figure S1).

The median age of the whole cohort at enrolment was 11.8 years (IQR, 6.6–13.8) (Table 1). There were 21 cohort studies, 12 randomised control trials and 1 case–control study, including 6866 participants and 18 different tests. Studies were published between 2005 and 2023. Four studies tested general populations; the rest tested CYP from high‐risk populations (first‐degree relatives or high‐risk on genetic testing [HLA‐conferred risk]). Twelve studies were conducted in the UK, 22 in Europe, 23 in North America and 9 in Oceania. Of the 34 studies, 12 were multi‐centre conducted in ≥4 countries. Twenty‐one studies included participants positive for multiple IA, whereas 13 had participants positive for ICA. Studies observed children over a median of 5 years (IQR, 2–5) to assess the occurrence of stage 3 type 1 diabetes, defined by the American Diabetes Association (*n* = 31) or the World Health Organisation criteria (*n* = 3).^6^, ^19^

**TABLE 1:** Summary of study characteristics for included studies (alphabetical order).

Study ID	Population study	Study type	Population type	Median age, (IQR)^a^	Participants, *n*	Index test(s) assessed
Studies which included participants with ≥2 islet autoantibodies						
Bediaga 2021 A	TrialNet (training)	Cohort	FDR	9.3 (6.2, 13.3)	1208	M120 DPTRS DPTRS60 Index60
Bediaga 2021 B	TrialNet (validation)	Cohort	FDR	9.9 (6.2, 14.5)	864	M120 DPTRS DPTRS60 Index60
Bediaga 2021 C	TEDDY	Cohort	HGR	6.6 (5.3, 7.9)	209	M120
Bediaga 2021 D	FR1DA	Cohort	GenPop	4.3 (3.3, 5.5)	80	M120
EldingLarsson 2015	DIAPREVENT	RCT	GenPop FDR	5.1 (4.0, 17.8)	49	OGTT IVGTT HbA1c C‐peptide HOMA‐IR
Felton 2022	TrialNet	Cohort	FDR	10.9 (7.0, 16.3)	6446	HOMA2‐B
Helminen 2015 A	DIPP	Cohort	HGR	3.3^a^ (2.6)	466	HbA1c
Helminen 2015 B	DIPP	Cohort	HGR	NR	908	OGTT Random glucose
Jacobsen 2019	TrialNet	Cohort	FDR	12.4 (9.39)	1815	Index60
Jacobsen 2022	TrialNet	Cohort	FDR	14.5 (10.4, 34.6)	2887	HbA1c Index60
Meah 2016	TrialNet	Cohort	FDR	11.9^a^ (7.5, 19.4)	1897	HOMA‐IR
Nathan 2017	TrialNet	Cohort	FDR	14.6^a^ (11.1)	1054	C‐peptide OGTT
Petruzelkova 2014	None	Cohort	FDR	NR	74	HbA1c
Salami 2022	TEDDY	Cohort	FDR HGR	NR	1033	HbA1c
Siljander 2013	DIPP	Cohort	HGR	6.4^b^	218	IVGTT
Sims 2022	TrialNet DPT‐1	Cohort	FDR	NR	1388	C‐peptide Index60
So 2022	TrialNet	Cohort	FDR	13.3^a^ (10.96)	3856	HbA1c C‐peptide HOMA‐IR Index60
Steck 2019	DAISY	Cohort	FDR HGR	NR	23	CGM
Truyen 2005	Belgian Diabetes Registry	Cohort	FDR	14 (8, 23)	338	PI:CP
VanDalem 2016	Belgian Diabetes Registry	Cohort	FDR	NR	49	HbA1c C‐peptide HOMA2‐IR PI:CP
Vehik 2022	TEDDY	Cohort	FDR HGR	3.6 (1.9–7.3)	1897	HbA1c OGTT
vonToerne 2017	BABYDIAB BABYDIET	Case control	FDR	3.2^b^	70	Peptide markers
Weiss 2022	FR1DA	Cohort	GenPop	4.1 (3.1, 5.4)	364	OGTT HbA1c PLS
Wilson 2023	TrialNet	Cohort	FDR	17.2 (11.7–36.4)	95	CGM
Studies which included participants with ICA positivity +/− direct diabetes autoantibody positivity						
Achenbach 2008	ENDIT	RCT	FDR	12.1 (8.4–18.1)	212	IVGTT
Barker 2007	DPT‐1	RCT	FDR	10.6 (7.11–14.13)	711	IVGTT OGTT
Beyan 2012	None	Cohort	GenPop	NR	115	CML
Bingley 2006	ENDIT	RCT	FDR	15.9 (10.4, 33.7)	549	OGTT IVGTT
Bingley 2008	ENDIT	RCT	FDR	11.7 (7.99–15.0)	213	OGTT IVGTT HOMA2‐IR
Ismail 2018	DPT‐1	RCT	FDR	13.8^a^ (9.6)	607	C‐peptide
Simmons 2020	DPT‐1	RCT	FDR	11.2 (3.0, 46.0)	654	OGTT DPTRS DPTRS60
Sosenko 2007	DPT‐1	RCT	FDR	13.9^a^ (9.6)	704	OGTT
Sosenko 2008	DPT‐1	RCT	FDR	13.8^a^ (9.6)	711	OGTT IVGTT C‐peptide
Sosenko 2009	DPT‐1	RCT	FDR	13.3^a^ (9.1)	515	OGTT
Sosenko 2015	DPT‐1	RCT	FDR	14.1^a^ (9.8)	670	C‐peptide
Xu 2007	DPT‐1	RCT	FDR	NR	356	HbA1c HOMA‐IR
Xu 2010	DPT‐1	RCT	FDR	NR	186	OGTT IVGTT

*Note*: A complete reference list for studies included in this review is shown in the supplementary.

Abbreviations: CML, serum N′‐carboxymethyl‐lysine; DPTRS, diabetes prevention trial risk score; FDR, First‐degree relative; GenPop, General Population; HbA1c, glycosylated haemoglobin; HGR, High genetic risk; HOMA‐IR/B, homeostatic model assessment for insulin resistance/beta‐cell function; IVGTT, intravenous glucose tolerance test; NR, not reported; OGTT, oral glucose tolerance test; RCT, randomised control trial.

^a^ Mean and standard deviation reported as median and interquartile range are not available.

^b^ Standard deviation/interquartile range not reported.

#### 3.1.1 Risk of bias assessment

Overall, most studies were at low risk of bias. Eleven studies were at high risk of bias due to a lack of a clear pre‐determined threshold in the index test. Five studies had an uncertain risk of bias (Table S4).

### 3.2 Test methods associated with progression to stage 3 type 1 diabetes

Four tests were associated with an increased risk of progression to stage 3 type 1 diabetes. Forest plots for tests not associated with progression are shown in the supplementary (Figures S2–S15), along with subgroup analyses (Figures S16–S56).

#### 3.2.1 Oral glucose tolerance test (fasting and 2‐h glucose)

**FIGURE 1:**
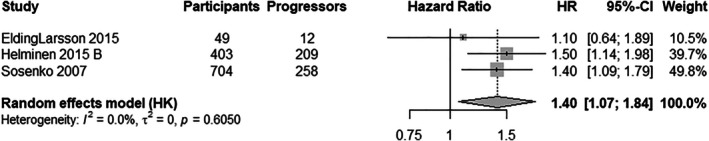
A random effects (HKSJ) meta‐analysis of studies showing the risk of developing type 1 diabetes per 1 mmol/L increase in fasting glucose during an OGTT.

A fasting glucose measured per 1 mmol/L increase was associated with an increased risk of progression (HR, 1.40; 95% CI, 1.07–1.84). Heterogeneity was low (*I* ^2^ = 0%; *p* = 0.61) (Figure 1).

Subgroup analyses by ICA, study intervention, risk of bias, risk period and population type showed no evidence of a difference in the pooled estimate or the level of heterogeneity (*p* = 0.98, *p* = 0.98, *p* = 0.51, *p* = 0.98, *p* = 0.61, respectively) (Figures S16–S20).

**FIGURE 2:**
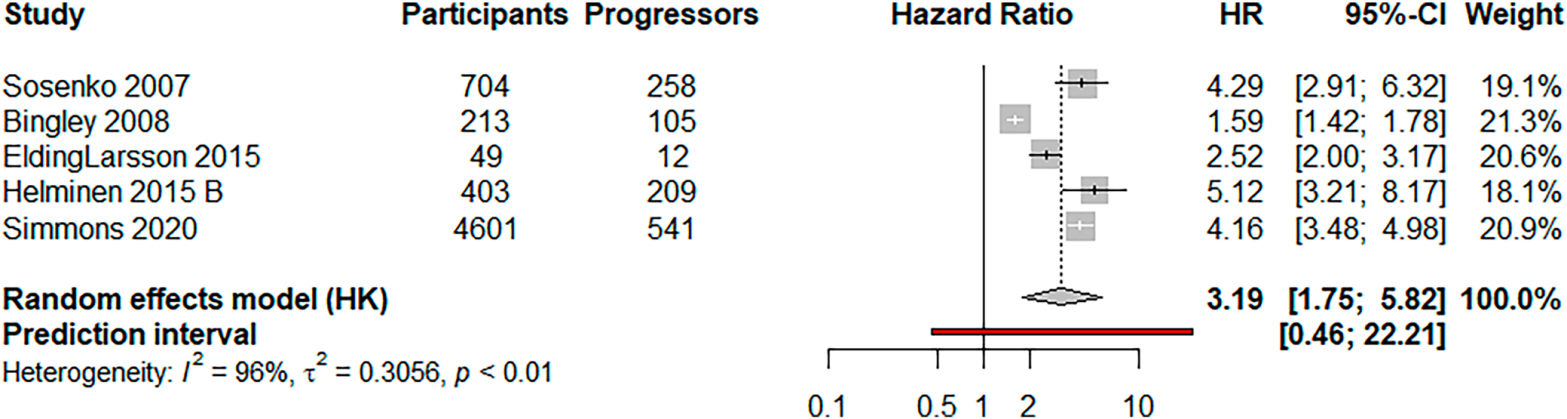
A random effects (HKSJ) meta‐analysis of studies showing the risk of developing type 1 diabetes following a 2‐hour glucose above the 75th centile during an OGTT.

A 2‐h glucose concentration above the 75th centile was associated with an increased risk of progression (HR, 3.19; 95% CI, 1.75–5.82) for all IA‐positive children (not separated by T1D stage). Heterogeneity was high, with a wide prediction interval (95% prediction interval, 0.46–22.21; *I* ^2^ = 96%; *p* < 0.01) (Figure 2).

Subgroup analyses by ICA, study intervention, risk period and population type showed no evidence of a difference in the pooled estimate (*p* = 0.77, *p* = 0.07, *p* = 0.77, *p* = 0.03, respectively). Studies at low risk of bias suggested that 2‐h glucose was more predictive (HR, 4.28; 95% CI, 3.52–5.19, *p* < 0.01). Heterogeneity fell to 0% when accounting for study intervention (Figures S21–S25).

#### 3.2.2 Glycated haemoglobin (HbA1c)

**FIGURE 3:**
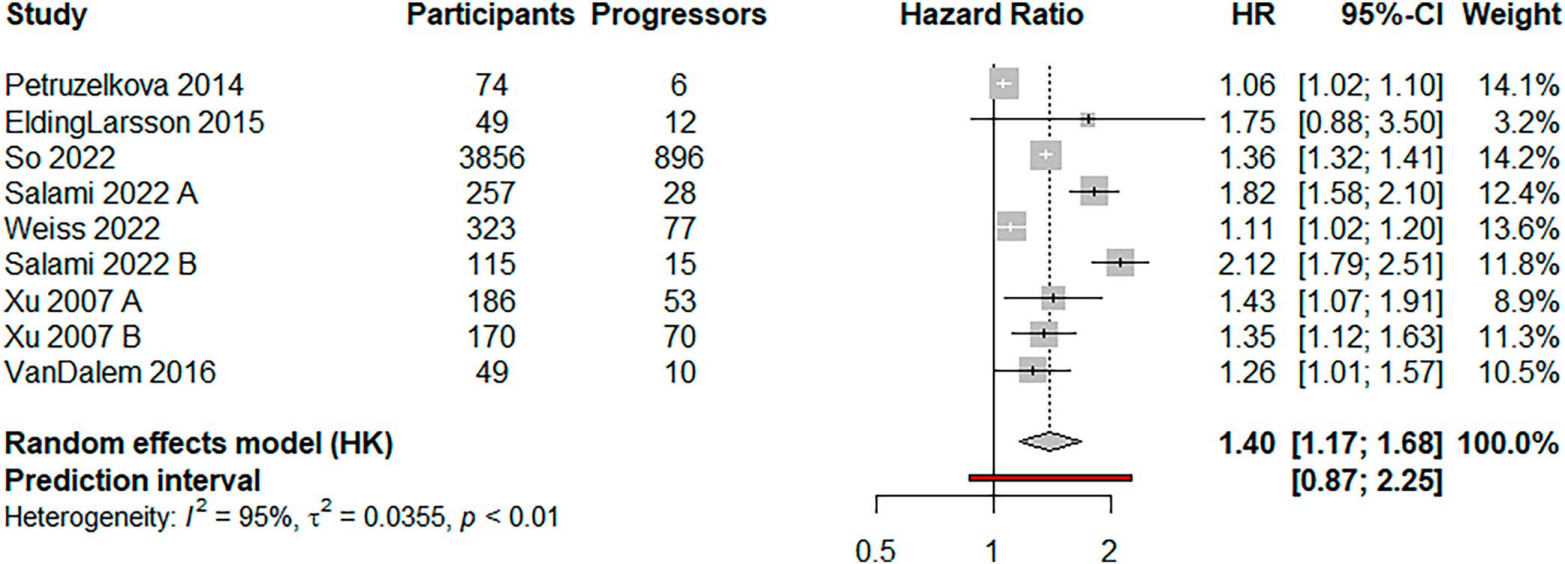
A random effects (HKSJ) meta‐analysis of studies showing the risk of developing type 1 diabetes per 1.1 mmol/mol (0.1%) increase in HbA1c.

HbA1c measured per 1.1 mmol/mol (0.1%) increase was associated with an increased risk of progression (HR, 1.40; 95% CI, 1.17–1.68). Heterogeneity was high, with a wide prediction interval (95% prediction interval, 0.87–2.25; *I* ^2^ = 95%; *p* < 0.01) (Figure 3).

Subgroup analyses by ICA and study intervention showed no evidence of a difference in the pooled estimate or the level of heterogeneity (*p* = 0.67 and *p* = 0.22, respectively). Studies that examined risk over 5 years or more showed that an increasing HbA1c was associated with a greater risk of progression (HR, 1.95; 95% CI, 0.74–5.12; *p* < 0.01). Studies with populations that included first‐degree relatives (FDR) and other genetically high‐risk CYP suggested that HbA1c was associated with a greater risk of progression (HR, 1.95; 95% CI, 0.74–5.12; *p* < 0.01). Studies at low risk of bias had low heterogeneity (*I* ^2^ = 0%) (Figures S26–S30).

#### 3.2.3 Risk progression scores

**FIGURE 4:**
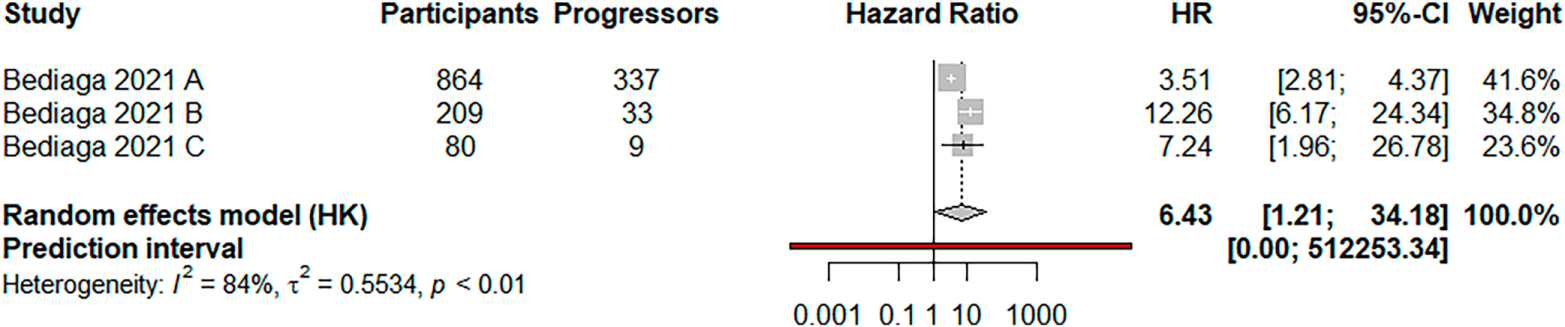
A random effects (HKSJ) meta‐analysis of studies showing the risk of developing type 1 diabetes following an M120 score above the median value.

One study combined three large datasets and was separated into Bediaga 2021 A (TrialNet), Bediaga 2021 B (TEDDY) and Bediaga 2021 C (FR1DA). An M120 score above the median was associated with an increased risk of progression (HR, 6.43; 95% CI, 1.21–34.18). Heterogeneity was high, with a very wide prediction interval (95% prediction interval, 0.00–512253.34; *I* ^2^ = 84%; *p* < 0.01) (Figure 4).

Subgroup analysis by risk period showed no evidence of an association with increased risk and low heterogeneity (HR, 3.74; 95% CI, 0.27–50.92; *I* ^2^ = 13%) (Figures S31 and S32).

Index60 analysis was separated into Sims 2022 A1 (TrialNet; change per year), Sims 2022 A2 (TrialNet; change per 6 months), Sims 2022 B1 (DPT‐1; change per year) and Sims 2022 B2 (DPT‐1; change per 6 months).

**FIGURE 5:**
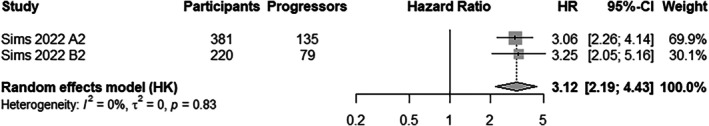
A random effects (HKSJ) meta‐analysis of studies showing the risk of developing type 1 diabetes per 1‐unit increase in Index60 over 6 months.

Index60 per 1‐unit increase over 6 months was associated with an increased risk of progression (HR, 3.12; 95% CI, 2.19–4.43). Heterogeneity was low (*I* ^2^ = 0%; *p* = 0.83) (Figure 5).

### 3.3 Test methods not associated with progression to stage 3 type 1 diabetes

#### 3.3.1 Oral glucose tolerance test (intermediate glucose)

There was no evidence that intermediate glucose (30‐ to 90‐min time points during an OGTT) measured per mmol/L increase was associated with progression (HR, 1.45; 95% CI, 0.32–6.46). Heterogeneity was low (*I* ^2^ = 7%; *p* = 0.30) (Figure S2).

#### 3.3.2 Oral glucose tolerance test (glucose area under the curve)

There was also no evidence that glucose AUC (sum of OGTT glucose values) above the 75th centile was associated with progression (HR, 4.39; 95% CI, 0.98–19.68). Heterogeneity was high, with a very wide prediction interval (95% prediction interval, 0.00–83665.28; *I* ^2^ = 98%; *p* < 0.01) (Figure S3).

Subgroup analyses by ICA, risk of bias, risk period and population type showed no evidence of an association with increased risk (*p* = 0.19, *p* = 0.51, *p* = 0.51, and *p* = 0.51, respectively). Studies that included an intervention suggested that glucose AUC was associated with an increased risk of progression (HR, 5.85; 95% CI, 2.28–15.02; *p* < 0.01) (Figures S33–S37).

#### 3.3.3 C‐peptide (fasting and stimulated)

There was no evidence that a fasting C‐peptide measured per 1 ng/mL decrease was associated with progression (HR, 2.06; 95% CI, 0.31–13.55). Heterogeneity was also high, with a wide prediction interval (95% prediction interval, 0.87–2.25; *I* ^2^ = 79%; *p* < 0.01) (Figure S4). There was also no evidence that a stimulated (30‐0‐min) C‐peptide measured per 1 ng/mL decrease was associated with progression (HR, 1.31; 95% CI, 0.75–2.29), with low heterogeneity (*I* ^2^ = 24%, *p* = 0.25) (Figure S5).

Subgroup analyses by ICA, study intervention, risk of bias (*p* = 0.23) and risk period (*p* = 0.58) showed no evidence of an association with fasting C‐peptide and progression, or the level of heterogeneity (Figures S38–S41).

#### 3.3.4 C‐peptide (area under the curve)

C‐peptide AUC analysis was separated into Sims 2022 A1 (TrialNet; change per year of C‐peptide AUC), Sims 2022 A2 (DPT‐1; change per year of C‐peptide AUC), Sims 2022 B1 (TrialNet; change per 6 months of C‐peptide AUC) and Sims 2022 B2 (DPT‐1; change per 6 months C‐peptide AUC).

There was no evidence that C‐peptide AUC measured per ng/ml decrease over 1 year was associated with progression (HR, 1.78; 95% CI, 0.20–15.91). Heterogeneity was high, with a wide prediction interval (95% prediction interval, 0.00–3448.98; *I* ^2^ = 94%; *p* < 0.01) (Figure S6).

There was also no evidence that a C‐peptide AUC measured per ng/ml decrease over 6 months was associated with progression (HR, 1.13; 95% CI, 0.88–1.45). Heterogeneity was low (*I* ^2^ = 0%; *p* = 0.37) (Figure S7).

There was no evidence of subgroup differences by study intervention, risk of bias, risk period and population type on the association of C‐peptide AUC measured over 6 or 12 months and progression (*p* = 0.38 [12 months], *p* = 0.79 [6 months]) (Figures S42–S49).

#### 3.3.5 Intravenous glucose tolerance test (IVGTT)

There was no evidence that first‐phase insulin response (FPIR) below the 10th centile measured during an IVGTT was associated with progression (HR, 1.02; 95% CI, 0.99–1.04). Heterogeneity was high, with a narrow prediction interval (95% prediction interval, 0.95–1.09; *I* ^2^ = 90%; *p* < 0.01) (Figure S8).

There was evidence of subgroup differences, showing FPIR below the 10th centile to be more predictive in studies that used defined antibodies and where participants were not randomised to an intervention (*p* < 0.01) and heterogeneity was reduced (*I* ^2^ = 0%) (Figures S50–S52).

#### 3.3.6 Continuous glucose monitoring (CGM)

There was no evidence that CGM measured per percentage time spent above 7.8 mmol/L (140 mg/dL) was associated with progression (HR, 1.09; 95% CI, 0.79–1.51), with low heterogeneity (*I* ^2^ = 0%; *p* = 1.00) (Figure S9).

There was also no evidence of an association between CGM and progression when measured per percentage time spent above 8.9 mmol/L (160 mg/dL) (HR, 1.06; 95% CI, 0.50–2.25). Heterogeneity was high (*I* ^2^ = 78%; *p* = 0.03) (Figure S10).

#### 3.3.7 Proinsulin to C‐peptide ratio (PI:CP)

There was no evidence that a PI:CP ratio above the 75th centile was associated with progression (HR, 3.22; 95% CI, 0.08–127.65). Heterogeneity was high (*I* ^2^ = 92%; *p* < 0.01) (Figure S11).

#### 3.3.8 Risk progression scores (Index60)

There was no evidence that Index60 measured per 1‐unit increase over 1 year was associated with progression with a high level of imprecision (HR, 6.35; 95% CI, 0.01–3616.53). Heterogeneity was also high (*I* ^2^ = 95%; *p* < 0.01) (Figure S12).

#### 3.3.9 Risk progression scores (HOMA‐IR)

One study reported data on HOMA‐IR from two separate cohorts in the DPT‐1 trial (Xu 2007 A [normoglycaemia, FPIR >10th centile] and Xu 2007 B [dysglycaemia, FPIR <10th centile]).

There was no evidence that HOMA‐IR measured per unit increase was associated with progression (HR, 1.48; 95% CI, 0.76–2.85). Heterogeneity was high, with a wide prediction interval (95% prediction interval, 0.27–8.00; *I* ^2^ = 76%; *p* < 0.01) (Figure S13).

There was no evidence of subgroup differences by ICA, study intervention, risk of bias and population type on the association of progression (*p* = 1.00, *p* = 0.08 and *p* = 0.28, respectively) (Figures S53–S56).

#### 3.3.10 Risk progression scores (HOMA2‐IR)

There was no evidence that HOMA2‐IR measured per unit increase was associated with progression with a high level of imprecision (HR, 7.20; 95% CI, 0.00–53291563158.76). Heterogeneity was also high (*I* ^2^ = 89%; *p* < 0.01) (Figure S14).

#### 3.3.11 Risk progression scores (HOMA2‐B)

Each study used the top tertile as a reference, with Felton 2022 A representing the middle tertile and Felton 2022 B representing the bottom tertile. There was no evidence that HOMA2‐B below the top tertile was associated with progression, with a high level of imprecision (HR, 1.82; 95% CI, 0.05–72.62). Heterogeneity was high (*I* ^2^ = 95%; *p* < 0.01) (Figure S15).

### 3.4 Studies that precluded meta‐analysis

Reasons for preclusion were data reported from a single study, or from different studies using the same population.

One study reported HbA1c change rather than absolute HbA1c level, precluding meta‐analysis. It showed evidence of an increased risk of progression to stage 3 type 1 diabetes after a 10% increase in HbA1c over 3–12 months (HR, 2.8; 95% CI, 2.0–4.1; *p* < 0.001) and two consecutive HbA1c results of 41 mmol/mol (5.9%) or more (HR, 8.5; 95% CI, 6.1–11.9; *p* < 0.001).^20^

One study showed that a random glucose of 7.8 mmol/L (140 mg/dL) or more was associated with an increased risk of progression (HR, 6.0; 95% CI, 4.3–8.6; *p* < 0.001).^21^ Another study showed that a sensor glucose (CGM) above 7.8 mmol/L (140 mg/dL) at least 10% of the time was associated with an increased risk of progression (HR, 2.39; 95% CI, 1.16–4.9).^22^

Two studies reporting data from the same cohort showed that a DPTRS above the median value (HR, 3.64; 95% CI, 2.93–4.52; *p* < 0.0001) or ≥7.26 (HR, 10.45; 95% CI, 8.80–12.41; *p* < 0.001) were associated with an increased risk of progression.^23^, ^24^

Two studies reporting data from the same cohort showed that a DPRTS60 above the median value (HR, 3.19; 95% CI, 2.57–3.96; *p* < 0.0001) or ≥4.93 (HR, 9.09; 95% CI, 7.66–10.78; *p* < 0.0001) were associated with an increased risk of progression (95% CI, 0.76–0.86).^23^, ^24^

One study showed a combined risk score (three protein biomarkers that assessed concentrations of three peptides [hepatocyte growth factor activator, complement factor H and ceruloplasmin] and age) was associated with an increased risk of progression for the high‐risk (HR, 3.94; 95% CI, 1.91–8.15) and medium‐risk groups (HR, 1.53; 95%, 0.77–3.07), using the low‐risk group as the comparator. The high‐risk group was also associated with an increased risk of progression compared to the medium‐risk group (HR, 2.38; 95% CI, 1.21–4.66).^25^

### 3.5 Reported diagnostic accuracy of test methods in predicting progression to stage 3 type 1 diabetes

Twenty‐two studies presented diagnostic accuracy data on 16 tests, using different thresholds for each test, preventing meta‐analysis. The M120 had the highest AUC under the ROC curve, with good precision (0.87, 0.79–0.94). CGM closely followed this, which also had a high AUC at two different thresholds, but the confidence intervals were wide and overlapped with other tests that reported AUC (0.85, 0.62–1.00). Of the other tests, HbA1c, fasting C‐peptide, HOMA‐IR, proinsulin to C‐peptide ratio and the OGTT all had similar AUCs around 0.7. Sensitivity and specificity depend upon the threshold chosen and fasting C‐peptide (<10th centile) had a high sensitivity (100%) with a corresponding low specificity (49%). The M120 (median value) also had a high sensitivity (91%), but a low specificity (58%). HbA1c had a lower sensitivity (70%), but a higher specificity (82%). Similarly, the progression likelihood score (PLS) also had a lower sensitivity (55%), but high specificity (94%). Several tests, including CGM, fasting glucose, 1‐h glucose and random plasma glucose, all had specificities above 90% (Table 2).

**TABLE 2:** Reported sensitivity and specificity of tests used to predict future stage 3 T1D onset.

Study ID	Threshold	Population type	Autoimmune status (≥ 2 IA or ICA)	Sensitivity	Specificity	PPV	NPV	AUC (95% CI)
Continuous glucose monitoring								
Steck 2019 A	18% time >7.8 mmol/L	FDR; HGR	≥2 IA	0.75	1.00	1.00	0.88	0.85 (0.62; 1.00)
Steck 2019 B	6% time >8.9 mmol/L	FDR; HGR	≥2 IA	0.625	0.93	0.83	0.82	0.85 (0.63; 1.00)
Carboxymethyl‐lysine								
Beyan 2012	600 ng/mL	Gen Pop	ICA	0.68	0.72	0.49	0.85	NR
Stimulated C‐peptide								
30‐ to 0‐min C‐peptide								
Ismail 2018 A	Median value	FDR	ICA	0.61	0.56	0.44	0.72	NR
120‐ to 60‐min C‐peptide								
Ismail 2018 B	Median value	FDR	ICA	0.62	0.56	0.44	0.73	NR
C‐peptide area under the curve								
Ismail 2018 C	Median value	FDR	ICA	0.60	0.56	0.43	0.71	NR
Fasting C‐peptide								
VanDalem 2016 C	<10th percentile	FDR	≥2 IA	1.00	0.49	0.33	1.00	0.78 (0.63; 0.93)
HbA1c								
Helminen 2015 A	10% increase over 3–12 months	HGR	≥2 IA	0.57	0.66	NR	NR	NR
VanDalem 2016 D	Not stated	FDR	≥2 IA	0.70	0.82	0.50	0.91	0.75 (0.57; 0.94)
HOMA2‐IR								
VanDalem 2016 B	Not stated	FDR	≥2 IA	0.80	0.59	0.33	0.92	0.74 (0.56; 0.92)
Intravenous glucose tolerance test								
Xu 2010 B	FPIR to HOMA‐IR ratio <49.22	FDR	ICA	0.68	0.61	0.41	0.83	0.66 (0.57; 0.74)
M120								
Trialnet training dataset								
Bediaga 2021 A	Median value	FDR	≥2 IA	0.77	0.57	0.31	0.91	0.75 (0.71; 0.79)
Trialnet validation dataset								
Bediaga 2021 B	Median value	FDR	≥2 IA	0.71	0.63	0.55	0.77	0.75 (0.71; 0.78)
TEDDY dataset								
Bediaga 2021 C	Median value	FDR	≥2 IA	0.91	0.58	0.29	0.97	0.87 (0.79; 0.94)
Fr1DA dataset								
Bediaga 2021 D	Median value	FDR	≥2 IA	0.89	0.56	0.20	0.98	0.74 (0.60; 0.88)
Oral glucose tolerance test								
Impaired fasting glucose								
Helminen 2015 B1	≥7 mmol/L	HGR	≥2 IA	0.06	0.98	0.76	0.49	NR
Xu 2010 C	≥4.9 mmol/L	FDR	ICA	0.47	0.56	0.31	0.72	0.49 (0.40; 0.59)
Impaired 2‐h glucose								
Helminen 2015 B2	≥7.8 mmol/L, <11.1 mmol/L	HGR	≥2 IA	0.35	0.95	0.88	0.58	NR
Xu 2010 A	≥6.3 mmol/L	FDR	ICA	0.62	0.71	0.46	0.83	0.67 (0.59; 0.76)
Simmons 2020 A	>7.8 mmol/L	FDR	ICA	0.79	0.67	0.04	0.99	0.71^a^
Vehik 2022	>8 mmol/L	FDR; HGR	≥2 IA	0.73	0.82	0.85	0.69	NR
Impaired 1‐h glucose								
Simmons 2020 B	>10 mmol/L	FDR	ICA	0.52	0.90	0.05	0.99	0.73^a^
Proinsulin to C‐peptide ratio								
VanDalem 2016 A	≥66th percentile	FDR	≥2 IA	0.70	0.77	0.44	0.91	0.68 (0.49; 0.87)
Progression likelihood score								
Weiss 2022	PLS score >4	Gen Pop	≥2 IA	0.55	0.94	0.50	0.95	NR
Random plasma glucose								
Helminen 2015 B3	≥7.8 mmol/L	HGR	≥2 IA	0.21	0.94	0.70	0.64	NR

Abbreviations: 95% CI, 95% confidence intervals; AUC, area under the curve; FDR, first degree relative; Gen Pop, general population; HGR, high HLA‐related genetic risk; IA, Islet autoantibodies; ICA, islet‐cell antibody; NPV, negative predictive value; PPV, positive predictive value; T1D, type 1 diabetes.

^a^ Confidence interval not reported.

## 4 DISCUSSION

This is the first systematic review and meta‐analysis to our knowledge that investigates the ability of tests to predict progression to stage 3 type 1 diabetes. We demonstrate that the fasting and 2‐h glucose of the OGTT, indices derived from it (M120, Index60) and HbA1c, can predict progression to stage 3, in CYP with early‐stage type 1 diabetes, using pooled HR data. Direct comparisons between tests should not be made, as tests were measured on different scales. Reported C‐statistics of ROC curves of these tests also suggest adequate diagnostic performance. Similarly, single studies showed that the DPTRS and DPTRS60 were associated with an increased risk of progression with good precision. These data highlight the consistent predictive performance of tests directly measuring glycaemia, with composite measures, such as the DPTRS attempting to build on the OGTT, or reduce the amount of sampling required (e.g. Index60, M120). There was insufficient evidence on the predictive value of intermediate glucose and glucose AUC during an OGTT, C‐peptide (fasting and stimulated), FPIR, CGM, PI:CP, HOMA‐IR, HOMA2‐IR and HOMA2‐B. This does not confirm the absence of the predictive value of negative tests, and therefore more definitive studies are needed where evidence was insufficient. Imprecision and heterogeneity were high for all but FPIR, which showed a modest non‐significant pooled estimate, with high precision, indicating marginal if any benefit from this test in predicting type 1 diabetes onset.

These findings are consistent with the standard staging of early‐stage type 1 diabetes in multiple IA‐positive individuals separating stage 1 (no dysglycaemia) from stage 2 (with dysglycaemia).^1^ Forty‐four percent of individuals with stage 1 vs. 75% with stage 2 progressed to stage 3 in 5 years. This is consistent with our finding on 2‐h glucose, but we also confirm that HbA1c and composites of glucose with C‐peptide (M120, Index‐60) are also predictive, indicating that several factors contribute to the risk of progression. Others have reported that the risk of progression is more nuanced than suggested by the staging classification, and recent data show that there is a large variation in progression rates between individuals in the same stage.^26^, ^27^

The strengths of this systematic review include the breadth of test methods included, facilitated by a comprehensive literature search and the inclusion of conference abstracts. Search‐related bias was minimised by using two independent reviewers for screening and not limiting the search by language. Potential sources of heterogeneity were assessed using subgroup analyses. Additionally, HKSJ methods were used to reduce type 1 error in the presence of high heterogeneity. The 95% prediction intervals were calculated to account for the heterogeneity and illustrate the likely range of values in future studies or clinical practice.

Limitations include the specific number and/or type of IA that could not be considered in the subgroup analyses, as this was not reported consistently. Having 3 or more IA, or IA‐2 positivity increases the risk of progression to stage 3 type 1 diabetes, so tests used in these subgroups would probably be more predictive, as it increases the pre‐test probability of progression.^27^, ^28^, ^29^ Further, the inclusion of children with single positive IA prevented the inclusion of some cohorts. This disproportionately affected newer test methods, for example, CGM, where data on children with multiple IA are combined with single IA data,^30^ thus reducing the pool of eligible studies compared with tests that have been studied over a longer period. Other studies assess CGM in the context of identifying and staging individuals with early‐stage type 1 diabetes,^31^, ^32^, ^33^ rather than progression, including adult populations,^34^, ^35^ further precluding inclusion. Studies adjusted estimates for different factors, which may have caused confounding. Pre‐determined thresholds were used, where raw data was not available. Therefore, we were unable to test alternative thresholds. The inconsistent reporting of study details prevented the investigation of potential confounders. It is possible that assay technology would have improved for the various tests over time, possibly benefiting more recent studies. Finally, overall levels of imprecision were high, likely because of insufficient studies for each test, with relatively small participant numbers for some studies.

Consensus recommendations have recently been published for monitoring individuals with early‐stage type 1 diabetes that provide alternative strategies to the OGTT.^7^, ^36^ Our systematic review adds evidence to the consensus, suggesting that whilst OGTT and OGTT‐derived indices are the appropriate gold standard for monitoring, the HbA1c is a practical and reliable alternative. Some individual studies have also suggested that CGM may provide a viable alternative to the OGTT, but this requires further evidence.^37^

The OGTT is an invasive test and can be impractical to perform, especially in young children.^38^, ^39^ A practical alternative approach derived from our results could be to reserve more invasive testing, such as the OGTT, for individuals who require more accurate monitoring, including those needing eligibility assessment for treatment or trial entry.

We provide evidence that CYP who progress to stage 3 type 1 diabetes can be identified using OGTT, OGTT‐derived indices (M120, Index60) and HbA1c. Further studies are needed, for example, CGM, and should include and report on populations with ≥2 IA.

## FUNDING INFORMATION

This study was supported by the Novo Nordisk UK Research Foundation, which funds RS through a research fellowship. REJB is funded by the NIHR Oxford Biomedical Research Centre for this work. KJB is funded through a research fellowship from JDRF Australia and Breakthrough T1D. PA is funded by NIHR Oxford Biomedical Research Centre, NIHR Oxford Health Biomedical Research Centre, NIHR Oxford and Thames Valley Applied Research Collaboration, and is an NIHR senior investigator. RAO receives funding from The Larry B and Leona M Helmsley Charitable Trust, Breakthrough T1D, NIH/NIDDK and Randox.

## CONFLICT OF INTEREST STATEMENT

RS has received speaker honoraria from Sanofi. REJB reports receiving speaker honoraria from Eli Lilly and Springer Healthcare, reports sitting on the Novo Nordisk UK Foundation Research Selection Committee voluntarily, acting as an independent advisor for Provent Bio, and received speaking honoraria from Sanofi and Medscape, which were donated to an education research fund. RAO reports receiving speaker honoraria for Sanofi and Novo Nordisk, Consulting for Sanofi, and research funding from Randox.

## Supporting information


Data S1.

## Data Availability

The data that support the findings of this study are available from the corresponding author upon reasonable request.
