# Supplementary material for: Testing methods used to predict disease progression in children with early‐stage type 1 diabetes: A systematic review and meta‐analysis
Source: Diabet Med. 2025 May 28;42(9):e70077. doi: 10.1111/dme.70077 (PMC12352723; doi:10.1111/dme.70077)
Supplement: Supplementary file 1 — Data S1. [file DME-42-e70077-s001.docx]

# SUPPLEMENTARY MATERIAL

## Tables

Supplementary Table 1. Full search strategy for the OVID Embase Database.

Supplementary Table 2. A summary of test methods used to predict progression to stage 3 type 1 diabetes, as described by included studies.

Supplementary Table 3. Details for calculating missing point estimates and rescaling or inverting point estimates.

Supplementary Table 4. Risk of bias assessment of included studies using QUADAS-2 tool.

Supplementary Table 5. Checklist of included items when reporting a systematic review and meta-analysis.

## Figures

Supplementary Figure 1. A PRISMA diagram detailing included and excluded studies.

Supplementary Figure 2-15. Forest plots showing meta-analyses of tests not associated with progression to stage 3 type 1 diabetes.

Supplementary Figures 16-56. Forest plots of subgroup analyses for each test group.

Supplementary Reference List: Reference list of the studies included in this systematic review and meta-analysis

Supplementary Table 1. Example of the full search strategy for Embase database, search updated on 07 August 2024.

| **#** | **Query** | **Results from 18 Jan 2023** |
| --- | --- | --- |
| 1 | exp child/ | 2,985,874 |
| 2 | exp adolescent/ | 1,722,451 |
| 3 | (infan* or newborn* or new-born* or perinat* or neonat* or baby* or babies or toddler* or minor* or boy* or girl* or kid or kids or child* or adolescen* or juvenil* or youth* or teen* or pubescen* or pediatric* or paediatric* or peadiatric* or school* or prematur* or preterm* or "pre term*" or prepub* or pre-pub* or preschool* or pre-school* or kindergarten* or nursery* or preadolescen* or pre-adolescen* or "young people" or "young person").ti,ab,kw. | 4,247,431 |
| 4 | 1 or 2 or 3 | 5,551,595 |
| 5 | insulin dependent diabetes mellitus/ | 134,649 |
| 6 | ("type 1 diabetes" or T1DM or T1D or "insulin dependent diabetes" or "juvenile onset diabetes" or IDDM or "autoimmune diabetes" or "auto-immune diabetes" or "ketosis-prone diabetes" or "sudden onset diabetes").ti,ab,kw. | 111,377 |
| 7 | 5 or 6 | 165,548 |
| 8 | early diagnosis/ | 123,731 |
| 9 | risk/ | 507,213 |
| 10 | (predict* or risk* or "early diagnos*" or progression or presymptomatic or pre-symptomatic or preclinical or pre-clinical).ti,ab,kw. | 6,925,607 |
| 11 | 8 or 9 or 10 | 7,011,972 |
| 12 | autoantibody/ | 80,892 |
| 13 | autoimmunity/ | 75,714 |
| 14 | genetic predisposition/ or genetic susceptibility/ | 195,308 |
| 15 | (autoantibod* or auto-antibod* or autoimmun* or auto-immun* or "genetic susceptib*" or "genetic predisposition*").ti,ab,kw. | 399,632 |
| 16 | 12 or 13 or 14 or 15 | 590,207 |
| 17 | mass screening/ or newborn screening/ | 82,087 |
| 18 | cohort analysis/ | 946,544 |
| 19 | (screening or monitoring or cohort* or longitudinal or follow* or prospective or surveillance).ti,ab,kw. | 8,670,931 |
| 20 | 17 or 18 or 19 | 8,807,026 |
| 21 | 4 and 7 and 11 and 16 and 20 | 2,472 |

Supplementary Table 2. A summary of test methods used to predict progression to stage 3 type 1 diabetes, as described by included studies.

| Index test | Metrics used | Comment |
| --- | --- | --- |
| CGM | % time spent above a glucose threshold | Measures interstitial glucose levels, taking a reading at regular intervals |
| CML | Serum CML above a predetermined threshold | An advanced glycation product, requires venous sampling |
| C-peptide | Fasting, 2-hour and intermediate timepoints (15, 30, 60, 90 minutes) | Typically requires venous sampling |
| DPTRS | Age, BMI, plasma glucose and C-peptide at 5 time points (0, 30, 60, 90, 120 minutes) | A composite risk score:  DPTRS = (1.569 * logBMI)  + (0.813 ∗ glucose sum from 30 to 120 mins)  - (0.848 ∗ C-peptide sum from 30 to 120 mins)  + (0.476 ∗ log fasting C-peptide) - (0.056 ∗ age) |
| DPTRS60 | Age, BMI, fasting plasma C-peptide, and plasma glucose and C-peptide at 60 minutes | A composite risk score:  DPTRS60 = (1.364 ∗ log BMI)  + (0.019 ∗ 60-min glucose)  - (0.311 ∗ 60-min C-peptide)  + (0.465 ∗ log fasting C-peptide) - (0.065 ∗ age) |
| HbA1c | A 10% rise over 3-12 months, or a level > 41 mmol/mol (5.9%) on consecutive samples | Requires plasma or capillary sampling. Represents glucose metabolism over a 2–3-month period. |
| HOMA-IR | Fasting insulin and glucose | A measure of insulin resistance.  HOMA-IR = fasting insulin (µU/ml) ∗ fasting glucose (mmol/L) / 22.5 |
| HOMA2-IR | Insulin and glucose | A measure of insulin resistance |
| HOMA2-B | Insulin and glucose | A measure of beta-cell function |
| Index60 | Fasting C-peptide, 60-minute glucose and C-peptide | A composite risk score:  Index60 = 0.3695 ∗ (log fasting C-peptide)  + 0.0165 ∗ (60-min glucose)  - 0.3644 ∗ (60-min C-peptide) |
| Intravenous glucose tolerance test | Plasma insulin | Requires venous insulin sampling to calculate FPIR  FPIR = sum of insulin at +1 and +3 mins. |
| M120 | Age, sex, BMI, HbA1c, IA-2 status, C-peptide and glucose at 120 minutes | A composite risk score:  M120 = 0.448 ∗ (sex) + 0.631 ∗ (IA-2A)  − 0.0302 ∗ (age) + 0.0605 ∗ (BMI)  + 1.380 ∗ (HbA1c) + 0.0265 ∗ (120 min glucose)  − 0.191 ∗ (120 min C-peptide) |
| OGTT | Plasma glucose at fasted, 2-hour post-glucose load, and intermediate timepoints | Requires venous sampling and an 8-hour minimum fast |
| Peptide markers | Age, hepatocyte growth factor activator, complement factor H, and ceruloplasmin | A composite measure of T1D risk |
| PI:CP | Proinsulin and C-peptide above a predetermined threshold | Requires venous sampling |
| Progression Likelihood Score | HbA1c, 90-minute plasma glucose, and islet-antigen positivity and titre | A composite risk score:  CPH model:  PLS = exp(HbA1c−5:233) ∗ 1.125  + (OGTT_90_−107.6) ∗ 0.0195  + (IA‐2A_cat_−1.27) ∗ 0.662 |
| Random plasma glucose | Plasma glucose above a predetermined threshold | Requires venous sampling, not timed with a meal. |

BMI, body mass index; CGM, continuous glucose monitoring; CML, N'-carboxymethyl-lysine; CPH, cox proportional hazards; DPTRS, diabetes prevention trial risk score; FPIR, first-phase insulin response; HbA1c, glycosylated haemoglobin; HOMA-IR/B, homeostatic model assessment for insulin resistance/beta-cell function; IA-2, insulinoma antigen-2; IVGTT, intravenous glucose tolerance test; OGTT, oral glucose tolerance test; PI:CP, proinsulin:C-peptide ratio.

Supplementary Table 3. Calculations for missing point estimates using log hazard ratio and p-value and rescaling or inverting point estimates.

| Step | Calculating missing point estimates using log hazard ratio (HR) and p-value |
| --- | --- |
| 1 | $\boldsymbol{Z=}\boldsymbol{\Phi}^{\boldsymbol{-1}}\boldsymbol{(1-}\frac{\text{p-value}}{\boldsymbol{2}}\boldsymbol{)}$  Φ^−1^ is the inverse cumulative distribution function of the standard normal distribution. |
| 2 | $\boldsymbol{S}\boldsymbol{E}_{\mathbf{log}\boldsymbol{(}\text{HR}\boldsymbol{)}}\boldsymbol{=}\frac{\mathbf{logHR}}{\boldsymbol{z} \boldsymbol{score}}$ |
| Step | Rescaling or inverting point estimates |
| 1 | $\boldsymbol{Z=}\frac{\text{x - }\text{µ}}{\boldsymbol{SD}}$ |
| 2 | Use a z-table to estimate the average of the upper quartile, or use “qnorm(75/100)” in R. Calculate summary descriptive statistics for normal distribution using R using “x<-rnorm(1000000)”, then “summary(x[x>qnorm(75/100)])”. |
| 3 | To calculate the logHR for a 1-SD change based on quartiles:  $\mathrm{logRR} \mathrm{for} a 1\mathrm{SD} \mathrm{difference}=B\times1=B=\frac{logRR4}{2.54}$  To calculate the logHR for quartiles based on a continuous variable per 1-SD:  𝑙𝑜𝑔𝑅𝑅4 = 2.54 × 𝑙𝑜𝑔H𝑅 for a 1-SD difference  If the point estimate is based on tertiles, convert the hazard ratio to quartiles equivalent:  $logRR4=B\times2.54=\frac{2.54}{2.18}\times logRR3$ |
| 4 | To rescale confidence intervals after recalculation of the logHR:  $\mathrm{SE}=\frac{2.54}{2.18} x \frac{(\mathrm{upperCI} ) - (\mathrm{lowerCI} )}{3.92}$ |

Supplementary Table 4. Risk of bias assessment of each domain for all studies using the QUADAS-2 tool.

|  | **Risk of Bias** | | | | **Applicability Concerns** | | |
| --- | --- | --- | --- | --- | --- | --- | --- |
| **Study ID** | **Patient selection** | **Index test** | **Reference standard** | **Flow and timing** | **Patient selection** | **Index test** | **Reference standard** |
| Achenbach 2008 | ☺ | ☺ | ☺ | 😐 | ☹ | 😐 | ☺ |
| Barker 2007 | ☺ | ☺ | ☺ | ☺ | ☹ | ☺ | ☺ |
| Bediaga 2021 | ☺ | ☺ | ☺ | ☺ | ☺ | ☺ | ☺ |
| Beyan 2012 | ☺ | ☹ | ☺ | ☺ | ☺ | ☺ | ☺ |
| Bingley 2006 | ☺ | ☺ | ☺ | ☺ | ☺ | ☺ | ☺ |
| Bingley 2008 | ☺ | 😐 | ☺ | ☺ | ☺ | ☺ | ☺ |
| Elding Larsson 2015 | ☺ | ☹ | ☺ | ☺ | ☺ | ☺ | ☺ |
| Felton 2022 | 😐 | ☹ | ☺ | ☺ | ☺ | ☺ | ☺ |
| Helminen 2015 A | ☺ | ☺ | ☺ | ☺ | ☺ | ☺ | ☺ |
| Helminen 2015 B | ☺ | ☺ | ☺ | ☺ | ☺ | ☺ | ☺ |
| Ismail 2018 | 😐 | ☹ | ☺ | ☺ | ☺ | ☺ | ☺ |
| Jacobsen 2019 | ☺ | ☺ | ☺ | ☺ | ☺ | ☺ | ☺ |
| Jacobsen 2022 | ☺ | ☺ | ☺ | ☺ | ☺ | ☺ | ☺ |
| Meah 2016 | ☺ | 😐 | ☺ | ☺ | ☺ | 😐 | ☺ |
| Nathan 2017 | ☺ | ☺ | ☺ | ☺ | ☺ | ☺ | ☺ |
| Petruzelkova 2014 | 😐 | ☹ | 😐 | 😐 | ☺ | ☺ | 😐 |
| Salami 2022 | ☺ | 😐 | 😐 | ☺ | ☺ | ☺ | ☺ |
| Siljander 2013 | ☺ | ☺ | ☺ | ☺ | ☺ | ☺ | ☺ |
| Simmons 2020 | 😐 | ☺ | ☺ | ☺ | ☺ | ☺ | ☺ |
| Sims 2022 | ☺ | ☺ | ☺ | ☺ | ☺ | ☺ | ☺ |
| So 2022 | ☺ | 😐 | ☺ | ☺ | ☺ | ☺ | ☺ |
| Sosenko 2007 | ☺ | ☺ | ☺ | ☺ | ☺ | ☺ | ☺ |
| Sosenko 2008 | ☺ | ☹ | ☺ | 😐 | ☺ | ☺ | ☺ |
| Sosenko 2009 | ☺ | ☺ | ☺ | ☺ | ☺ | ☺ | ☺ |
| Sosenko 2015 | 😐 | ☹ | ☺ | 😐 | ☹ | ☺ | ☺ |
| Steck 2019 | ☺ | ☹ | ☺ | ☺ | ☺ | ☺ | ☺ |
| Truyen 2005 | ☹ | ☹ | ☺ | ☺ | ☹ | ☺ | ☺ |
| Van Dalem 2016 | ☺ | ☺ | ☺ | ☺ | ☺ | ☺ | ☺ |
| Vehik 2022 | ☺ | ☺ | ☺ | ☺ | ☺ | ☺ | ☺ |
| von Toerne 2017 | ☹ | 😐 | ☺ | ☺ | ☺ | ☺ | ☺ |
| Weiss 2022 | ☺ | ☹ | ☺ | ☺ | ☺ | ☺ | ☺ |
| Wilson 2023 | ☺ | ☺ | ☺ | ☺ | ☺ | ☺ | ☺ |
| Xu 2007 | ☺ | ☺ | ☺ | ☺ | ☺ | ☺ | ☺ |
| Xu 2010 | ☺ | ☹ | ☺ | ☹ | ☺ | ☺ | ☺ |

Supplementary Table 5. A checklist of included items reported for this systematic review and meta-analyses.

| **Section and Topic** | **Item #** | **Checklist item** | **Location where item is reported** |
| --- | --- | --- | --- |
| **TITLE** | | | |
| Title | 1 | Identify the report as a systematic review and/or meta-analysis. | Title page |
| **ABSTRACT** | | | |
| Abstract | 2 | Structured summary provided as appropriate: background, objectives, data sources, study selection, eligibility, study appraisal and synthesis methods, results limitations, conclusions and implications, registration number. | Abstract |
| **INTRODUCTION** | | | |
| Rationale | 3 | Describe the rationale for the review in the context of existing knowledge. | Introduction paragraph 2 |
| Objectives | 4 | Provide an explicit statement of the objective(s) or question(s) the review addresses. | Introduction paragraph 2 |
| **METHODS** | | | |
| Eligibility criteria | 5 | Specify the inclusion and exclusion criteria for the review and how studies were grouped for the syntheses. | Methods paragraphs 2, 6 and 7 |
| Information sources | 6 | Specify all databases, registers, websites, organisations, reference lists and other sources searched or consulted to identify studies. Specify the date when each source was last searched or consulted. | Methods paragraph 1 and supplementary table 1 |
| Search strategy | 7 | Present the full search strategies for all databases, registers and websites, including any filters and limits used. | Supplementary table 1 |
| Selection process | 8 | Specify the methods used to decide whether a study met the inclusion criteria of the review, including how many reviewers screened each record and each report retrieved, whether they worked independently, and if applicable, details of automation tools used in the process. | Methods paragraph 3 |
| Data collection process | 9 | Specify the methods used to collect data from reports, including how many reviewers collected data from each report, whether they worked independently, any processes for obtaining or confirming data from study investigators, and if applicable, details of automation tools used in the process. | Methods paragraph 3 and 4 |
| Data items | 10a | List and define all outcomes for which data were sought. Specify whether all results that were compatible with each outcome domain in each study were sought (e.g. for all measures, time points, analyses), and if not, the methods used to decide which results to collect. | Methods paragraph 4 |
|  | 10b | List and define all other variables for which data were sought (e.g. participant and intervention characteristics, funding sources). Describe any assumptions made about any missing or unclear information. | Methods paragraphs 4 and 6 |
| Study risk of bias assessment | 11 | Specify the methods used to assess risk of bias in the included studies, including details of the tool(s) used, how many reviewers assessed each study and whether they worked independently, and if applicable, details of automation tools used in the process. | Methods paragraph 5 |
| Effect measures | 12 | Specify for each outcome the effect measure(s) (e.g. risk ratio, mean difference) used in the synthesis or presentation of results. | Methods paragraph 4 |
| Synthesis methods | 13a | Describe the processes used to decide which studies were eligible for each synthesis (e.g. tabulating the study intervention characteristics and comparing against the planned groups for each synthesis (item #5)). | Methods paragraphs 4, 6 and 7 |
|  | 13b | Describe any methods required to prepare the data for presentation or synthesis, such as handling of missing summary statistics, or data conversions. | Methods paragraphs 4 and 6 |
|  | 13c | Describe any methods used to tabulate or visually display the results of individual studies and syntheses. | Methods paragraph 5 |
|  | 13d | Describe any methods used to synthesize results and provide a rationale for the choice(s). If meta-analysis was performed, describe the model(s), method(s) to identify the presence and extent of statistical heterogeneity, and software package(s) used. | Methods paragraph 5 |
|  | 13e | Describe any methods used to explore possible causes of heterogeneity among study results (e.g. subgroup analysis, meta-regression). | Methods paragraph 6 |
|  | 13f | Describe any sensitivity analyses conducted to assess robustness of the synthesized results. | Methods paragraph 6 |
| Reporting bias assessment | 14 | Describe any methods used to assess risk of bias due to missing results in a synthesis (arising from reporting biases). | Methods paragraph 6 |
| Certainty assessment | 15 | Describe any methods used to assess certainty (or confidence) in the body of evidence for an outcome. | Methods paragraph 5 |
| **RESULTS** | | | |
| Study selection | 16a | Describe the results of the search and selection process, from the number of records identified in the search to the number of studies included in the review, ideally using a flow diagram. | Results paragraph 1 and 2 |
|  | 16b | Cite studies that might appear to meet the inclusion criteria, but which were excluded, and explain why they were excluded. | Supplementary Figure 1 |
| Study characteristics | 17 | Cite each included study and present its characteristics. | Supplementary table 1 |
| Risk of bias in studies | 18 | Present assessments of risk of bias for each included study. | Results paragraph 38 |
| Results of individual studies | 19 | For all outcomes, present, for each study: (a) summary statistics for each group (where appropriate) and (b) an effect estimate and its precision (e.g. confidence/credible interval), ideally using structured tables or plots. | Results Figure 1A/B, 2A/B and Supplementary Figures 2-15 |
| Results of syntheses | 20a | For each synthesis, briefly summarise the characteristics and risk of bias among contributing studies. | Results Figure 1A/B, 2A/B, Supplementary Figures 2-45 and Supplementary Table 3. |
|  | 20b | Present results of all statistical syntheses conducted. If meta-analysis was done, present for each the summary estimate and its precision (e.g. confidence/credible interval) and measures of statistical heterogeneity. If comparing groups, describe the direction of the effect. | Results Figure 1A/B, 2A/B and Supplementary Figures 2-45 |
|  | 20c | Present results of all investigations of possible causes of heterogeneity among study results. | Supplementary Figures 16-45 |
|  | 20d | Present results of all sensitivity analyses conducted to assess the robustness of the synthesized results. | N/A |
| Reporting biases | 21 | Present assessments of risk of bias due to missing results (arising from reporting biases) for each synthesis assessed. | N/A |
| Certainty of evidence | 22 | Present assessments of certainty (or confidence) in the body of evidence for each outcome assessed. | Conclusions paragraph 1 |
| **DISCUSSION** | | | |
| Discussion | 23a | Provide a general interpretation of the results in the context of other evidence. | Conclusions paragraph 1 |
|  | 23b | Discuss any limitations of the evidence included in the review. | Conclusions paragraph 3 |
|  | 23c | Discuss any limitations of the review processes used. | Conclusions paragraph 3 |
|  | 23d | Discuss implications of the results for practice, policy, and future research. | Conclusions paragraph 4 and 5 |
| **OTHER INFORMATION** | | | |
| Registration and protocol | 24a | Provide registration information for the review, including register name and registration number, or state that the review was not registered. | Methods paragraph 1 |
|  | 24b | Indicate where the review protocol can be accessed, or state that a protocol was not prepared. | Methods paragraph 1 |
|  | 24c | Describe and explain any amendments to information provided at registration or in the protocol. | N/A |
| Support | 25 | Describe sources of financial or non-financial support for the review, and the role of the funders or sponsors in the review. | Funding paragraph 1 |
| Competing interests | 26 | Declare any competing interests of review authors. | Conflicts of interest statement paragraph 1 |
| Availability of data, code and other materials | 27 | Report which of the following are publicly available and where they can be found: template data collection forms; data extracted from included studies; data used for all analyses; analytic code; any other materials used in the review. | N/A |

Supplementary Figure 1. A PRISMA diagram showing the flow of included studies.

### Forest plots of tests not associated with progression to stage 3 type 1 diabetes

Supplementary Figure 2. A random-effects (HKSJ) meta-analysis of studies showing the risk of type 1 diabetes per mmol/L higher intermediate glucose during an OGTT.

Supplementary Figure 3. A random-effects (HKSJ) meta-analysis of studies showing the risk of type 1 diabetes following a glucose area under the curve above the 75^th^ centile during an OGTT.

Supplementary Figure 4. A random-effects (HKSJ) meta-analysis of studies showing the risk developing type 1 diabetes per ng/ml lower fasting C-peptide level.

Supplementary Figure 5. A random-effects (HKSJ) meta-analysis of studies showing the risk of developing type 1 diabetes per ng/ml lower stimulated (30-0 minute) C-peptide level.

Supplementary Figure 6. A random-effects (HKSJ) meta-analysis of studies showing the risk of developing type 1 diabetes per ng/ml decrease in C-peptide AUC per year following an oral glucose tolerance test.

Supplementary Figure 7. A random-effects (HKSJ) meta-analysis of studies showing the risk of developing type 1 diabetes per ng/ml decrease in C-peptide AUC per 6 months following an oral glucose tolerance test.

Supplementary Figure 8. A random-effects (HKSJ) meta-analysis showing the risk of type 1 diabetes based on a first-phase insulin response below the 10^th^ centile, during an intravenous glucose tolerance test.

Supplementary Figure 9. A random-effects (HKSJ) meta-analysis showing the risk of type 1 diabetes based on % time spent above 7.8mmol/L (140mg/dl) using a continuous glucose monitor.

Supplementary Figure 10. A random-effects (HKSJ) meta-analysis showing the risk of type 1 diabetes based on % time spent above 8.9mmol/L (160mg/dl) using a continuous glucose monitor.

Supplementary Figure 11. A random-effects (HKSJ) meta-analysis showing the risk of type 1 diabetes based on a pro-insulin to C-peptide ratio above the 75^th^ centile.

Supplementary Figure 12. A random-effects (HKSJ) meta-analysis showing the risk of developing type 1 diabetes per 1-unit increase in Index60 over 12 months.

Supplementary Figure 13. A random-effects (HKSJ) meta-analysis of studies showing the risk of developing type 1 diabetes per 1-unit increase in HOMA-IR.

Supplementary Figure 14. A random-effects (HKSJ) meta-analysis of studies showing the risk of developing type 1 diabetes per 1-unit increase in HOMA2-IR.

Supplementary Figure 15. A random-effects (HKSJ) meta-analysis of studies showing the risk of developing type 1 diabetes with a HOMA2-B below the top tertile.

### Forest plots of subgroup analyses for each test group

### OGTT (Fasting glucose)

Supplementary Figure 16. A subgroup random-effects (HKSJ) meta-analysis of studies showing the risk of developing type 1 diabetes per 1 mmol/L fasting glucose increase during an OGTT, categorised by the use of ICA as an inclusion criterion for participants.

Supplementary Figure 17. A subgroup random-effects (HKSJ) meta-analysis of studies showing the risk of developing type 1 diabetes per 1 mmol/L fasting glucose increase during an OGTT, categorised based on the use of an intervention.

Supplementary Figure 18. A subgroup random-effects (HKSJ) meta-analysis of studies showing the risk of developing type 1 diabetes per 1 mmol/L fasting glucose increase during an OGTT, categorised based on risk of bias.

Supplementary Figure 19. A subgroup random-effects (HKSJ) meta-analysis of studies showing the risk of developing type 1 diabetes per 1 mmol/L fasting glucose increase during an OGTT, categorised based on risk period.

Supplementary Figure 20. A subgroup random-effects (HKSJ) meta-analysis of studies showing the risk of developing type 1 diabetes per 1 mmol/L fasting glucose increase during an OGTT, categorised based on population.

### OGTT (2-hour)

Supplementary Figure 21. A subgroup random-effects (HKSJ) meta-analysis of studies showing the risk of developing type 1 diabetes after a 2-hour glucose above the 75^th^ centile during an OGTT, categorised by the use of ICA as an inclusion criterion for participants.

Supplementary Figure 22. A subgroup random-effects (HKSJ) meta-analysis of studies showing the risk of developing type 1 diabetes after a 2-hour glucose above the 75^th^ centile during an OGTT, categorised based on the use of an intervention.

Supplementary Figure 23. A subgroup random-effects (HKSJ) meta-analysis of studies showing the risk of developing type 1 diabetes after a 2-hour glucose above the 75^th^ centile during an OGTT, categorised based on risk of bias.

Supplementary Figure 24. A subgroup random-effects (HKSJ) meta-analysis of studies showing the risk of developing type 1 diabetes after a 2-hour glucose above the 75^th^ centile during an OGTT, categorised based on risk period.

Supplementary Figure 25. A subgroup random-effects (HKSJ) meta-analysis of studies showing the risk of developing type 1 diabetes after a 2-hour glucose above the 75^th^ centile during an OGTT, categorised based on population.

### HbA1c

Supplementary Figure 26. A subgroup random-effects (HKSJ) meta-analysis of studies showing the risk of developing type 1 diabetes per 1.1 mmol/mol increased in HbA1c, categorised by the use of ICA as an inclusion criterion for participants.

Supplementary Figure 27. A subgroup random-effects (HKSJ) meta-analysis of studies showing the risk of developing type 1 diabetes per 1.1 mmol/mol increase in HbA1c, categorised by the use of an intervention.

Supplementary Figure 28. A subgroup random-effects (HKSJ) meta-analysis of studies showing the risk of developing type 1 diabetes per 1.1 mmol/mol increase in HbA1c, categorised by risk of bias.

Supplementary Figure 29. A subgroup random-effects (HKSJ) meta-analysis of studies showing the risk of developing type 1 diabetes per 1.1 mmol/mol increase in HbA1c, categorised by reported risk period.

Supplementary Figure 30. A subgroup random-effects (HKSJ) meta-analysis of studies showing the risk of developing type 1 diabetes per 1.1 mmol/mol increase in HbA1c, categorised by population type.

### M120

Supplementary Figure 31. A sub-group random-effects (HKSJ) meta-analysis of studies showing the risk of developing type 1 diabetes after an M120 above the median value, categorised by reported risk period.

Supplementary Figure 32. A sub-group random-effects (HKSJ) meta-analysis of studies showing the risk of developing type 1 diabetes after an M120 above the median value, categorised by population type.

### OGTT (AUC)

Supplementary Figure 33. A subgroup random-effects (HKSJ) meta-analysis of studies showing the risk of developing type 1 diabetes after an area under the curve glucose above the 75^th^ centile during an OGTT, categorised by the use of ICA as an inclusion criterion for participants.

Supplementary Figure 34. A subgroup random-effects (HKSJ) meta-analysis of studies showing the risk of developing type 1 diabetes after an area under the curve glucose above the 75^th^ centile during an OGTT, categorised based on the use of an intervention.

Supplementary Figure 35. A subgroup random-effects (HKSJ) meta-analysis of studies showing the risk of developing type 1 diabetes after an area under the curve glucose above the 75th centile during an OGTT, categorised based on risk of bias.

Supplementary Figure 36. A subgroup random-effects (HKSJ) meta-analysis of studies showing the risk of developing type 1 diabetes after an area under the curve glucose above the 75^th^ centile during an OGTT, categorised based on risk period.

Supplementary Figure 37. A subgroup random-effects (HKSJ) meta-analysis of studies showing the risk of developing type 1 diabetes after an area under the curve glucose above the 75th centile during an OGTT, categorised based on population.

### C-peptide (Fasting)

Supplementary Figure 38. A subgroup random-effects (HKSJ) meta-analysis of studies showing the risk of developing type 1 diabetes per ng/ml decrease in fasting C-peptide level, categorised by the use of ICA as an inclusion criterion for participants.

Supplementary Figure 39. A subgroup random-effects (HKSJ) meta-analysis of studies showing the risk of developing type 1 diabetes per ng/ml decrease in fasting C-peptide level, categorised by the use of an intervention.

Supplementary Figure 40. A subgroup random-effects (HKSJ) meta-analysis of studies showing the risk of developing type 1 diabetes per ng/ml decrease in fasting C-peptide level, categorised by risk of bias.

Supplementary Figure 41. A subgroup random-effects (HKSJ) meta-analysis of studies showing the risk of developing type 1 diabetes per ng/ml decrease in fasting C-peptide level, categorised by reported risk period.

### C-peptide (AUC)

Supplementary Figure 42. A subgroup random-effects (HKSJ) meta-analysis of studies showing the risk of developing type 1 diabetes per ng/ml decrease in C-peptide AUC per year following an oral glucose tolerance test, categorised by the use of an intervention.

Supplementary Figure 43. A subgroup random-effects (HKSJ) meta-analysis of studies showing the risk of developing type 1 diabetes per ng/ml decrease in C-peptide AUC per year following an oral glucose tolerance test, categorised by risk of bias.

Supplementary Figure 44. A subgroup random-effects (HKSJ) meta-analysis of studies showing the risk of developing type 1 diabetes per ng/ml decrease in C-peptide AUC per year following an oral glucose tolerance test, categorised by reported risk period.

Supplementary Figure 45. A subgroup random-effects (HKSJ) meta-analysis of studies showing the risk of developing type 1 diabetes per ng/ml decrease in C-peptide AUC per year following an oral glucose tolerance test, categorised by population type.

Supplementary Figure 46. A subgroup random-effects (HKSJ) meta-analysis of studies showing the risk of developing type 1 diabetes per ng/ml decrease in C-peptide AUC per 6 months following an oral glucose tolerance test, categorised by the use of an intervention.

Supplementary Figure 47. A subgroup random-effects (HKSJ) meta-analysis of studies showing the risk of developing type 1 diabetes per ng/ml decrease in C-peptide AUC per 6 months following an oral glucose tolerance test, categorised by risk of bias.

Supplementary Figure 48. A subgroup random-effects (HKSJ) meta-analysis of studies showing the risk of developing type 1 diabetes per ng/ml decrease in C-peptide AUC per 6 months following an oral glucose tolerance test, categorised by reported risk period.

Supplementary Figure 49. A subgroup random-effects (HKSJ) meta-analysis of studies showing the risk of developing type 1 diabetes per ng/ml decrease in C-peptide AUC per 6 months following an oral glucose tolerance test, categorised by population type.

### IVGTT (FPIR)

Supplementary Figure 50. A subgroup random-effects (HKSJ) meta-analysis showing the risk of developing type 1 diabetes based on a first-phase insulin response below the 10^th^ centile, during an intravenous glucose tolerance test, categorised by the use of ICA as an inclusion criterion for participants.

Supplementary Figure 51. A random-effects (HKSJ) meta-analysis showing the risk of developing type 1 diabetes based on a first-phase insulin response below the 10^th^ centile, during an intravenous glucose tolerance test, categorised by the use of an intervention.

Supplementary Figure 52. A random-effects (HKSJ) meta-analysis showing the risk of developing type 1 diabetes based on a first-phase insulin response below the 10^th^ centile, during an intravenous glucose tolerance test, categorised by risk of bias.

### HOMA-IR

Supplementary Figure 53. A sub-group (HKSJ) random-effects meta-analysis of studies showing the risk of developing type 1 diabetes after an abnormal HOMA-IR, categorised using ICA as an inclusion criterion for participants.

Supplementary Figure 54. A sub-group (HKSJ) random-effects meta-analysis of studies showing the risk of developing type 1 diabetes after an abnormal HOMA-IR, categorised by the inclusion of an intervention.

Supplementary Figure 55. A sub-group (HKSJ) random-effects meta-analysis of studies showing the risk of developing type 1 diabetes after an abnormal HOMA-IR, categorised by risk of bias.

Supplementary Figure 56. A sub-group (HKSJ) random-effects meta-analysis of studies showing the risk of developing type 1 diabetes after an abnormal HOMA-IR, categorised by population type.

### Supplementary Reference List: (1–34); studies included in this systematic review and meta-analysis.

1.	Bediaga NG, Li-Wai-Suen CSN, Haller MJ, Gitelman SE, Evans-Molina C, Gottlieb PA, Hippich M, Ziegler AG, Lernmark A, DiMeglio LA, Wherrett DK, Colman PG, Harrison LC, Wentworth JM. Simplifying prediction of disease progression in pre-symptomatic type 1 diabetes using a single blood sample. *Diabetologia*. 2021;64(11).

2.	Elding Larsson H, Larsson C, Lernmark Å. Baseline heterogeneity in glucose metabolism marks the risk for type 1 diabetes and complicates secondary prevention. *Acta Diabetol*. 2015;52(3):473-481.

3.	Felton JL, Cuthbertson D, Warnock M, Lohano K, Meah F, Wentworth JM, Sosenko J, Evans-Molina C. HOMA2-B enhances assessment of type 1 diabetes risk among TrialNet Pathway to Prevention participants. *Diabetologia*. 2022;65(1):88-100.

4.	Helminen O, Aspholm S, Pokka T, Hautakangas MR, Haatanen N, Lempainen J, Ilonen J, Simell O, Knip M, Veijola R. HbA1c Predicts Time to Diagnosis of Type 1 Diabetes in Children at Risk. *Diabetes*. 2015;64(5):1719-1727.

5.	Helminen O, Aspholm S, Pokka T, Ilonen J, Simell O, Veijola R, Knip M. OGTT and random plasma glucose in the prediction of type 1 diabetes and time to diagnosis. *Diabetologia*. 2015;58(8):1787-1796.

6.	Jacobsen LM, Bocchino L, Evans-Molina C, DiMeglio L, Goland R, Wilson DM, Atkinson MA, Aye T, Russell WE, Wentworth JM, Boulware D, Geyer S, Sosenko JM. The risk of progression to type 1 diabetes is highly variable in individuals with multiple autoantibodies following screening. *Diabetologia*. 2020;63(3):588-596.

7.	Jacobsen LM, Bundy BN, Ismail HM, Clements M, Warnock M, Geyer S, Schatz DA, Sosenko JM. Index60 Is Superior to HbA1c for Identifying Individuals at High Risk for Type 1 Diabetes. *J Clin Endocrinol Metab*. 2022;107(10):2784-2792.

8.	Meah FA, DiMeglio LA, Greenbaum CJ, Blum JS, Sosenko JM, Pugliese A, Geyer S, Xu P, Evans-Molina C. The relationship between BMI and insulin resistance and progression from single to multiple autoantibody positivity and type 1 diabetes among TrialNet Pathway to Prevention participants. *Diabetologia*. 2016;59(6):1186-1195.

9.	Nathan BM, Boulware D, Geyer S, Atkinson MA, Colman P, Goland R, Russell W, Wentworth JM, Wilson DM, Evans-Molina C, Wherrett D, Skyler JS, Moran A, Sosenko JM, Type 1 Diabetes TrialNet and Diabetes Prevention Trial–Type 1 Study Groups. Dysglycemia and Index60 as Prediagnostic End Points for Type 1 Diabetes Prevention Trials. *Diabetes Care*. 2017;40(11):1494-1499.

10.	Petruzelkova L, Vcelakova J, Labikova J, Lebl J, Kolouskova S. HbA1c Level as a Predictive Marker of Progression to Clinical Diabetes. ESPE Abstracts. 2014. <https://abstracts.eurospe.org/hrp/0082/hrp0082fc3.2>. Accessed March 25, 2024.

11.	Salami F, Tamura R, You L, Lernmark Å, Larsson HE, Lundgren M, Krischer J, Ziegler A, Toppari J, Veijola R, Rewers M, Haller MJ, Hagopian W, Akolkar B, Törn C. HbA1c as a time predictive biomarker for an additional islet autoantibody and type 1 diabetes in seroconverted TEDDY children. *Pediatr Diabetes*. 2022;23(8):1586-1593.

12.	Siljander HT, Hermann R, Hekkala A, Lähde J, Tanner L, Keskinen P, Ilonen J, Simell O, Veijola R, Knip M. Insulin secretion and sensitivity in the prediction of type 1 diabetes in children with advanced β-cell autoimmunity. *Eur J Endocrinol*. 2013;169(4):479-485.

13.	Sims EK, Cuthbertson D, Felton JL, Ismail HM, Nathan BM, Jacobsen LM, Paprocki E, Pugliese A, Palmer J, Atkinson M, Evans-Molina C, Skyler JS, Redondo MJ, Herold KC, Sosenko JM. Persistence of β-Cell Responsiveness for Over Two Years in Autoantibody-Positive Children With Marked Metabolic Impairment at Screening. *Diabetes Care*. 2022;45(12):2982-2990.

14.	So M, O’Rourke C, Ylescupidez A, Bahnson HT, Steck AK, Wentworth JM, Bruggeman BS, Lord S, Greenbaum CJ, Speake C. Characterising the age-dependent effects of risk factors on type 1 diabetes progression. *Diabetologia*. 2022;65(4):684-694.

15.	Steck AK, Dong F, Taki I, Hoffman M, Simmons K, Frohnert BI, Rewers MJ. Continuous Glucose Monitoring Predicts Progression to Diabetes in Autoantibody Positive Children. *J Clin Endocrinol Metab*. 2019;104:3337-3344.

16.	Truyen I, De Pauw P, Jørgensen PN, Van Schravendijk C, Ubani O, Decochez K, Vandemeulebroucke E, Weets I, Mao R, Pipeleers DG, Gorus FK. Proinsulin levels and the proinsulin:c-peptide ratio complement autoantibody measurement for predicting type 1 diabetes. *Diabetologia*. 2005;48(11):2322-2329.

17.	Van Dalem A, Demeester S, Balti E V., Keymeulen B, Gillard P, Lapauw B, De Block C, Abrams P, Weber E, Vermeulen I, De Pauw P, Pipeleers D, Weets I, Gorus FK. Prediction of Impending Type 1 Diabetes through Automated Dual-Label Measurement of Proinsulin:C-Peptide Ratio. Nishimura W, ed. *PLoS One*. 2016;11(12):e0166702.

18.	Vehik K, Boulware D, Killian M, Rewers M, McIndoe R, Toppari J, Lernmark Å, Akolkar B, Ziegler AG, Rodriguez H, Schatz DA, Krischer JP, Hagopian W, Rewers M, Barbour A, Bautista K, Baxter J, Felipe-Morales D, Frohnert BI, Stahl M, Gesualdo P, Hoffman M, Karban R, Liu E, Munoz A, Norris J, O’Donnell H, Peacock S, Shorrosh H, Steck A, Stern M, Waugh K, Toppari J, Simell OG, Adamsson A, Aaltonen SM, Ahonen S, Åkerlund M, Hakola L, Hekkala A, Holappa H, Hyöty H, Ikonen A, Ilonen J, Jokipuu S, Karlsson L, Kero J, Koskenniemi JJ, Kähönen M, Knip M, Koivikko ML, Kokkonen K, Koskinen M, Koreasalo M, Kurppa K, Kuusela S, Kytölä J, Laiho J, Latva-aho T, Leppänen L, Lindfors K, Lönnrot M, Mäntymäki E, Mattila M, Miettinen M, Multasuo K, Mykkänen T, Niininen T, Niinistö S, Nyblom M, Oikarinen S, Ollikainen P, Othmani Z, Pohjola S, Rautanen J, Riikonen A, Romo M, Simell S, Tossavainen P, Vähä-Mäkilä M, Varjonen E, Veijola R, Viinikangas I, Virtanen SM, She JX, Schatz D, Hopkins D, Steed L, Bryant J, Silvis K, Haller M, Gardiner M, McIndoe R, Sharma A, Anderson StephenW, Jacobsen L, Marks J, Towe PD, et al. Rising Hemoglobin A1c in the Nondiabetic Range Predicts Progression of Type 1 Diabetes As Well As Oral Glucose Tolerance Tests. *Diabetes Care*. 2022;45(10):2342-2349.

19.	von Toerne C, Laimighofer M, Achenbach P, Beyerlein A, de las Heras Gala T, Krumsiek J, Theis FJ, Ziegler AG, Hauck SM. Peptide serum markers in islet autoantibody-positive children. *Diabetologia*. 2017;60(2):287-295.

20.	Weiss A, Zapardiel-Gonzalo J, Voss F, Jolink M, Stock J, Haupt F, Kick K, Welzhofer T, Heublein A, Winkler C, Achenbach P, Ziegler AG, Bonifacio E. Progression likelihood score identifies substages of presymptomatic type 1 diabetes in childhood public health screening. *Diabetologia*. 2022;65(12).

21.	Wilson DM, Pietropaolo SL, Acevedo-Calado M, Huang S, Anyaiwe D, Scheinker D, Steck AK, Vasudevan MM, McKay S V, Sherr JL, Herold KC, Dunne JL, Greenbaum CJ, Lord SM, Haller MJ, Schatz DA, Atkinson MA, Nelson PW, Pietropaolo M. CGM Metrics Identify Dysglycemic States in Participants From the TrialNet Pathway to Prevention Study. *Diabetes Care*. 2023;46(3):526-534.

22.	Achenbach P, Bonifacio E, Williams AJK, Ziegler AG, Gale EAM, Bingley PJ, ENDIT Group. Autoantibodies to IA-2beta improve diabetes risk assessment in high-risk relatives. *Diabetologia*. 2008;51(3):488-492.

23.	Barker JM, McFann K, Harrison LC, Fourlanos S, Krischer J, Cuthbertson D, Chase HP, Eisenbarth GS, DPT-1 Study Group. Pre-type 1 diabetes dysmetabolism: maximal sensitivity achieved with both oral and intravenous glucose tolerance testing. *J Pediatr*. 2007;150(1):31-36.e6.

24.	Beyan H, Riese H, Hawa MI, Beretta G, Davidson HW, Hutton JC, Burger H, Schlosser M, Snieder H, Boehm BO, Leslie RD. Glycotoxin and Autoantibodies Are Additive Environmentally Determined Predictors of Type 1 Diabetes. *Diabetes*. 2012;61(5):1192-1198.

25.	Bingley PJ, Gale EAM. Progression to type 1 diabetes in islet cell antibody-positive relatives in the European Nicotinamide Diabetes Intervention Trial: the role of additional immune, genetic and metabolic markers of risk. *Diabetologia*. 2006;49(5):881-890.

26.	Bingley PJ, Mahon JL, Gale EAM. Insulin Resistance and Progression to Type 1 Diabetes in the European Nicotinamide Diabetes Intervention Trial (ENDIT). *Diabetes Care*. 2008;31(1):146-150.

27.	Ismail HM, Becker DJ, Libman I, Herold KC, Redondo MJ, Atkinson MA, Cleves MA, Palmer J, Sosenko J. Early and late C‐peptide responses during oral glucose tolerance testing are oppositely predictive of type 1 diabetes in autoantibody‐positive individuals. *Diabetes Obes Metab*. 2020;22(6):997-1000.

28.	Simmons KM, Sosenko JM, Warnock M, Geyer S, Ismail HM, Elding Larsson H, Steck AK. One-Hour Oral Glucose Tolerance Tests for the Prediction and Diagnostic Surveillance of Type 1 Diabetes. *J Clin Endocrinol Metab*. 2020;105(11):e4094-e4101.

29.	Sosenko JM, Palmer JP, Greenbaum CJ, Mahon J, Cowie C, Krischer JP, Chase HP, White NH, Buckingham B, Herold KC, Cuthbertson D, Skyler JS. Increasing the Accuracy of Oral Glucose Tolerance Testing and Extending Its Application to Individuals With Normal Glucose Tolerance for the Prediction of Type 1 Diabetes. *Diabetes Care*. 2007;30(1):38-42.

30.	Sosenko JM, Krischer JP, Palmer JP, Mahon J, Cowie C, Greenbaum CJ, Cuthbertson D, Lachin JM, Skyler JS. A Risk Score for Type 1 Diabetes Derived From Autoantibody-Positive Participants in the Diabetes Prevention Trial–Type 1. *Diabetes Care*. 2008;31(3):528-533.

31.	Sosenko JM, Palmer JP, Rafkin-Mervis L, Krischer JP, Cuthbertson D, Mahon J, Greenbaum CJ, Cowie CC, Skyler JS. Incident Dysglycemia and Progression to Type 1 Diabetes Among Participants in the Diabetes Prevention Trial–Type 1. *Diabetes Care*. 2009;32(9):1603-1607.

32.	Sosenko JM, Skyler JS, Dimeglio LA, Beam CA, Krischer JP, Greenbaum CJ, Boulware D, Rafkin LE, Matheson D, Herold KC, Mahon J, Palmer JP. A new approach for diagnosing type 1 diabetes in autoantibody-positive individuals based on prediction and natural history. *Diabetes Care*. 2015;38(2).

33.	Xu P, Cuthbertson D, Greenbaum C, Palmer JP, Krischer JP. Role of Insulin Resistance in Predicting Progression to Type 1 Diabetes. *Diabetes Care*. 2007;30(9):2314-2320.

34.	Xu P, Wu Y, Zhu Y, Dagne G, Johnson G, Cuthbertson D, Krischer JP, Sosenko JM, Skyler JS. Prognostic Performance of Metabolic Indexes in Predicting Onset of Type 1 Diabetes. *Diabetes Care*. 2010;33(12):2508-2513.
